# Transcranial Direct Current Stimulation (tDCS) of the visual cortex: a proof-of-concept study based on interictal electrophysiological abnormalities in migraine

**DOI:** 10.1186/1129-2377-14-23

**Published:** 2013-03-11

**Authors:** Alessandro Viganò, Tullia Sasso D’Elia, Simona Liliana Sava, Maurie Auvé, Victor De Pasqua, Alfredo Colosimo, Vittorio Di Piero, Jean Schoenen, Delphine Magis

**Affiliations:** 1 Headache Research Unit. Dept of Neurology, University of Liège, Liege, Belgium; 2 Giga-Neurosciences, University of Liège, Liege, Belgium; 3 Brain Morphometry and Dynamics Lab. Dept of Anatomy, Histology, Forensic Medicine and Orthopaedics, Sapienza, Sapienza University of Rome, Rome, Italy; 4 Neurology A. Dept Of Neurology and Psychiatry, Sapienza University of Rome, Rome, Italy

**Keywords:** Migraine, Habituation deficit, tDCS, Treatment, Visual cortex

## Abstract

**Background:**

Preventive pharmacotherapy for migraine is not satisfactory because of the low efficacy/tolerability ratio of many available drugs. Novel and more efficient preventive strategies are therefore warranted. Abnormal excitability of cortical areas appears to play a pivotal role in migraine pathophysiology. Transcranial direct current stimulation (tDCS) is a non-invasive and safe technique that is able to durably modulate the activity of the underlying cerebral cortex, and is being tested in various medical indications. The results of small open studies using tDCS in migraine prophylaxis are conflicting, possibly because the optimal stimulation settings and the brain targets were not well chosen. We have previously shown that the cerebral cortex, especially the visual cortex, is hyperresponsive in migraine patients between attacks and provided evidence from evoked potential studies that this is due to a decreased cortical preactivation level. If one accepts this concept, anodal tDCS over the visual cortex may have therapeutic potentials in migraine prevention, as it is able to increase neuronal firing.

**Objective:**

To study the effects of anodal tDCS on visual cortex activity in healthy volunteers (HV) and episodic migraine without aura patients (MoA), and its potentials for migraine prevention.

**Methods:**

We recorded pattern-reversal visual evoked potentials (VEP) before and after a 15-min session of anodal tDCS over the visual cortex in 11 HV and 13 MoA interictally. Then 10 MoA patients reporting at least 4 attacks/month subsequently participated in a therapeutic study, and received 2 similar sessions of tDCS per week for 8 weeks as migraine preventive therapy.

**Results:**

In HV as well as in MoA, anodal tDCS transiently increased habituation of the VEP N1P1 component. VEP amplitudes were not modified by tDCS. Preventive treatment with anodal tDCS turned out to be beneficial in MoA: migraine attack frequency, migraine days, attack duration and acute medication intake significantly decreased during the treatment period compared to pre-treatment baseline (all p < 0.05), and this benefit persisted on average 4.8 weeks after the end of tDCS.

**Conclusions:**

Anodal tDCS over the visual cortex is thus able to increase habituation to repetitive visual stimuli in healthy volunteers and in episodic migraineurs, who on average lack habituation interictally. Moreover, 2 weekly sessions of anodal tDCS had a significant preventive anti- migraine effect, proofing the concept that the low preactivation level of the visual cortex in migraine patients can be corrected by an activating neurostimulation. The therapeutic results indicate that a larger sham-controlled trial using the same tDCS protocol is worthwhile.

## Background

Finding the ‘right’ migraine preventive treatment often remains a challenge in many patients. The drugs currently used in migraine prophylaxis (such as antiepileptics, beta blockers…) are not migraine-specific, unlike acute therapies like triptans or gepans, which were designed to treat headache. Moreover, they are not devoid of side-effects and their efficacy rarely exceeds 50-60% for the best of them [1]. Chronic migraine patients, i.e. the presence of at least 15 days of headache per month, of which at least 8 migraine attacks, represent almost the 2-3% of the population and they are particularly difficult to manage as their response to existing preventive therapies is often unsatisfactory [2,3]. There is thus a need for new effective and well-tolerated treatments in migraine prophylaxis. The latter should ideally be more disease-specific, i.e. designed to counteract the dysfunctions known to be involved in migraine pathogenesis.

Migraine is a complex and heterogeneous disorder, in which genetics and environment interact to generate dysfunctioning paths and loops at several levels of the central nervous system. These intricate phenomena are responsible for the multifaceted clinical features of the disease and especially its dynamics characterized by a cyclic ictal-interictal pattern and the repetition of attacks [4].

It has been known for a long time that the brain excitability is abnormal in migraine during the interictal period [5]. However many past studies on cortical excitability had provided conflicting results, and whether the brain was hyperexcitable [6-8] or hypoexcitable [9-12] remained extensively debated for years. A recent theory proposed a semantic modification that was able to unify these opposite hypotheses, i.e. that the brain cortex was not hyper*excitable* per se but hyper*responsive* to sensory stimuli in migraine between attacks [13]. A reproducible hallmark mirroring this hyperresponsiveness is the lack of habituation to repeated sensory or cognitive stimulations reported in both evoked potentials and neuroimaging trials (for review, see [14]). Habituation is defined as a behavioural response decrement that results from repeated stimulations and does not involve sensory adaptation or fatigue, i.e. a decrease in peripheral receptor activity [15]. According to Groves and Thompson, habituation relies on the balance of two opposite mechanisms, facilitation and depression of brain responses to a sensory stimulus.

In the interictal period of migraine, many evoked potentials studies to various sensory modalities found on average lower initial response amplitudes followed by a decreased habituation -or even a potentiation- of subsequent responses, whereas in healthy subjects a higher initial response preceded a more pronounced habituation. These results paved the way to the hypothesis that the lack of habituation was possibly due to a lower preactivation level of brain sensory cortices, according to the ceiling theory [16]. Recent studies suggested that this lower preactivation level could be the consequence of impaired functional thalamocortical loops, the so-called Thalamocortical Dysrhythmia, a dysfunction, which is also involved in other neurological diseases [17,18]. Further works demonstrated that the lack of habituation was not constant and normalized just before and during the migraine attack. Interestingly, it was recently shown that in chronic migraine patients habituation was normal [19] but evolved to potentiation when these chronic migraineurs went to remission towards episodic migraine [20], suggesting that chronic migraine could be considered as a “never-ending attack” [21].

In the last decade there has been an increasing interest for neuromodulation in migraine treatment [22]. Even if randomized controlled trials are scarce, some preliminary results are encouraging and peripheral and central neuromodulating techniques are considered as promising alternatives to pharmacological treatment. Among them, 2 central non-invasive techniques appear particularly suitable for migraine preventive treatment: repetitive transcranial magnetic stimulation (rTMS) and trancranial Direct Current Stimulation (tDCS). Both are able to durably modify the excitability of the underlying cortex and could potentially correct the functional abnormalities found in migraine patients. They were already applied in several other neurological diseases with some success [23]. High frequency (around 10 Hz) rTMS stimulation can increase brain excitability, while low frequency rTMS (about 1 Hz) is able to decrease it [23,24]. Anodal tDCS appears to increase brain excitability, while cathodal tDCS stimulation decreases it [23,25-27] though not all studies agreed on this point [28].

Few recent therapeutic trials applied rTMS and tDCS in migraine prevention, and their results were conflicting [29-31]. This could be due to dissimilarities in their stimulation protocols, as stimulated brain regions as well as stimulation frequencies, length and intensities were different and depended on the baseline pathophysiological hypothesis, mainly the belief that the migrainous brain was hyperexcitable or, on the contrary, hypoexcitable. Moreover, these trials did not assess the brain excitability before and after treatment. In a previous study, we had reported that a single 10 Hz excitatory rTMS session was able to restore normal habituation and initial amplitude of visual-evoked responses (VEPs) in migraineurs, and that this effect lasted at least 9 minutes. In a subsequent trial, this stimulation was applied on 5 successive days, but the VEPs normalization did not exceed several hours in most migraineurs. However, these results had not been applied in a preventative therapeutic study for now, and whether the normalization of habituation was associated to a clinical improvement remained unknown [12,32].

We therefore performed a pilot proof-of-concept study combining the two approaches for the first time, but we used anodal (i.e. excitatory) tDCS instead of 10 Hz-rTMS. This was a 2-step trial: we first repeated the electrophysiological study in healthy volunteers and migraineurs in order to ensure that anodal tDCS could modulate habituation and correct the impaired interictal excitability in migraineurs like rTMS, then in the second phase the same stimulation paradigm was converted into a preventive therapy for episodic migraine in a prospective pilot trial.

## Methods

1. Subjects and clinical records

Eleven healthy volunteers (HV) were enrolled for the electrophysiological study (5 males and 6 females, mean age 25.8 ± SD 5.7 years). Exclusion criteria were: age below 18 or above 65 years, a personal history of recurrent headache or other neurological diseases especially seizures, familial history of recurrent headache, child migraine equivalents (motion sickness, cyclic vomiting or recurrent abdominal pain, somnambulism etc.…), chronic pain syndromes, analgesics intake at the time of recording, and contra-indications to tDCS neurostimulation (metal prosthetics in the head or internal stimulation like a pacemaker). They were compared to 13 migraineurs without aura (MoA) according to the second International Classification of Headache Disorders (ICHD-IIR) criteria (2 males and 11 females, mean age 29.3 ± 5.1). Patients had more than 2 and less than 8 attacks/month and were not under preventive therapy for at least 3 months before the experimental day. All volunteers and patients were naive to any kind of neurostimulation, i.e. they never got this type of treatment before (central or peripheral neurostimulation), whatever the indication was. Patients were recruited in the outpatient clinic through headache-specialized consultations (DM and JS).

The therapeutic study involved 10 migraineurs suffering from episodic MoA (2 males and 8 females, mean age 38.4 ± 16.3) with a frequency ranging between 3 and 8 attacks/month, knowing that none of them fulfilled the criteria for chronic migraine. Only two of them were previously involved in the electrophysiological study. Intake of a drug preventive treatment was allowed in the therapeutic study only, but this pharmacological therapy had to be stable for at least 2 months. Five out of the 10 enrolled patients were under preventive therapy at the moment of the trial: one was taking riboflavin alone, two riboflavin associated with a beta-blocker (metoprolol or propranolol), the other two were under topiramate. All of them had treatment for several months and this treatment did not give them any satisfaction. The average time under prophylactic therapy at inclusion was 3.2 months (2 patients were under preventive therapy for 2 months, the other 3 for 4 months).

During the whole therapeutic study period the patients were asked to fill a headache diary to record migraine attacks, migraine and headache days, pain intensity in a scale from 1 (light) to 3 (severe), duration of attack (hours), medication intake, and associated symptoms (nausea, vomiting, photo- and phonophobia). This headache diary had to be completed at least 2 months before the treatment initiation, in order to have a 2-month pretreatment baseline.

All subjects participating in the electrophysiological and/or the therapeutic studies received detailed oral and written explanations of the whole experiment provided by the experimenter (AV or TSD) and gave written informed consent. This study was approved by the local Ethics Committee of the CHR Citadelle Hospital of Liège, Belgium.

2. Material and stimulation protocols

### Electrophysiological study

For the electrophysiological study we recorder pattern reversal visual evoked potentials (PR-VEPs), as described before [33]. PR-VEPs were selected as they are one of the best studied electrophysiological responses in migraine, where a decreased preactivation level and a lack of habituation has been reported in many studies [34]. Briefly, subjects sat in a comfortable armchair in a quite dark room at a +/− 90 cm distance from the monitor. They were asked to relax and to fix a red sticker in the centre of the screen (Nicolet™; 24 × 18 cm) with their right eye, the left eye being covered by a patch. The visual stimulus was a checkerboard pattern of black and white squares (15 mm side, 80% contrast, mean luminance 250 cd/m^2^, colour temperature 9500 K) alternating at a frequency of 3.1 Hz. Pin-electrodes were used to record the signal: the active electrode was inserted at Oz and was referenced to Fz according to the 10–20 system [34]. The ground electrode was fixed to the right forearm. During uninterrupted stimulation, 600 cortical responses were recorded (CED™ 1902 preamplifier and CED™ Micro1401 converter; Cambridge Electronic Design Ltd, Cambridge, UK). Two hundred and fifty milliseconds of the poststimulus period were sampled at a rate of 4000 Hz.

Acquisitions were made at baseline (T0), immediately after (T1) and 3 hours after (T2) a single anodal tDCS session (see below). At the end of the first VEPs recording (T0), the place of the pin electrodes was marked with a pen, in order to ensure that their locations remained the same in the subsequent recordings (T1 and T2). Hence, after T1 the subjects had a 3-hour free time before coming back to the laboratory for T2 acquisition. During this period, they were not allowed to smoke, to drink alcohol or beverages containing caffeine or other energy drinks, and to take a nap. All recordings were distant from at least 72 hours of a migraine attack. The time of the last attack was checked on patient’s diary and the absence of an attack occurrence within the next 72 hours after the experiment was checked by phone call. To avoid changes of cortical excitability due to hormonal variations, all female subjects performed the experiment in the first half of the menstrual cycle.

### Anodal tDCS

Anodal tDCS stimulation was performed using a programmable DC stimulator (NeuroConn, Ilmeanu, Germany^**©**^) with 2 rubber electrodes (5x7cm). The anode was placed in the occipital region near Oz in order to stimulate the underlying visual cortex, and the cathode was fixed on the chin. We chose to put the cathode outside the cranial vault in order to avoid a concomitant inhibition of other cerebral cortices, for example the frontal cortex when Fz had been chosen as cathode. The subjects were stimulated at 1 mA intensity and each session lasted 15 minutes. To decrease their possible discomfort the stimulation increased gradually during the first 8 seconds and decreased progressively within the last 8 seconds of the tDCS.

Thus, the electrophysiological study comprised a single tDCS session and in the therapeutic pilot study anodal tDCS was applied twice a week for 8 weeks, i.e. 16 sessions, using the same tDCS parameters. The 2 weekly sessions were fixed, i.e. were always applied the same days during the whole treatment period of a single patient (for example, every Tuesday and Friday).

3. Data analysis and statistics.

**Figure 1:**
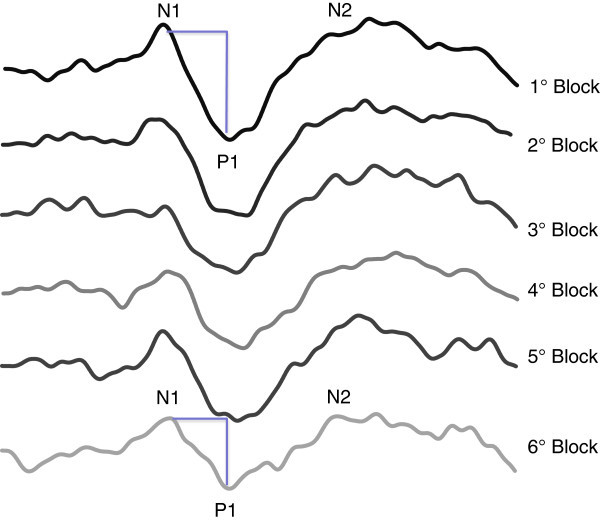
**The time-dependent changes of N1P1 and P1N2 components of visual evoked potentials in a healthy subject.** Over six blocks of 100 averaged single trial responses a reduction in amplitude of both components is shown, in the representative example.

In the electrophysiological study, the 600 PR-VEP responses were averaged off-line into six blocks of 100 responses using Signal™ software version 4 (Cambridge Electronic Design Ltd, bandpass 1–100 Hz). The peak-to-peak N1–P1 and P1-N2 amplitudes were measured, N1 being the most negative point around 70 ms latency after the stimulus (range 60–90), P1 the most positive around 100 ms latency (range 80–130) and N2 the most negative point following P1 between 90 and 200 ms. To visualize better the slope of N1P1 and P1N2 amplitude changes over the total duration of visual stimulation, a linear regression analysis of the mean amplitudes in the 6 blocks of 100 averages responses was performed and considered as the reflect of habituation degree (see Figure 1). Hence, a normal habituation gave a negative slope value, while potentiation gave a positive slope. We calculated means and standard deviations for the first block amplitude (first 100 averaged N1P1 VEP responses, ìV, which reflects cortical preactivation level – see above introduction) and N1P1 and P1N2 habituation slopes, at T0, T1 and T2, and compared them between HV and MoA.

In the therapeutic study we followed prospectively the evolution of migraine attack frequency, migraine days, mean pain intensity, attack duration and acute drugs intake during treatment with tDCS, compared to the baseline. We compared baseline clinical variables (2^nd^ month) with those of the 2nd month of tDCS treatment, to study the cumulative effect of the repeated stimulation.

Statistical calculations were carried out using STATISTICA (version 7, StatSoft, Oklahoma, USA). We first used the Shapiro-Wilk test to assess the distribution of the variables. Since most of them did not fit the normal distribution, we used Wilcoxon signed-rank test (paired samples) to study modifications induced over time by tDCS within the same subjects, and we employed Whitney–Mann U-test to compare electrophysiological values between HV and MoA groups. The time-dependent changes in habituation were assessed with one-way analysis of variance (ANOVA) for repeated measures. We also did a post-hoc comparison with Wilcoxon signed-rank test. All results were considered significant at p < 0.05.

## Results

### Electrophysiological study

**Figure 2:**
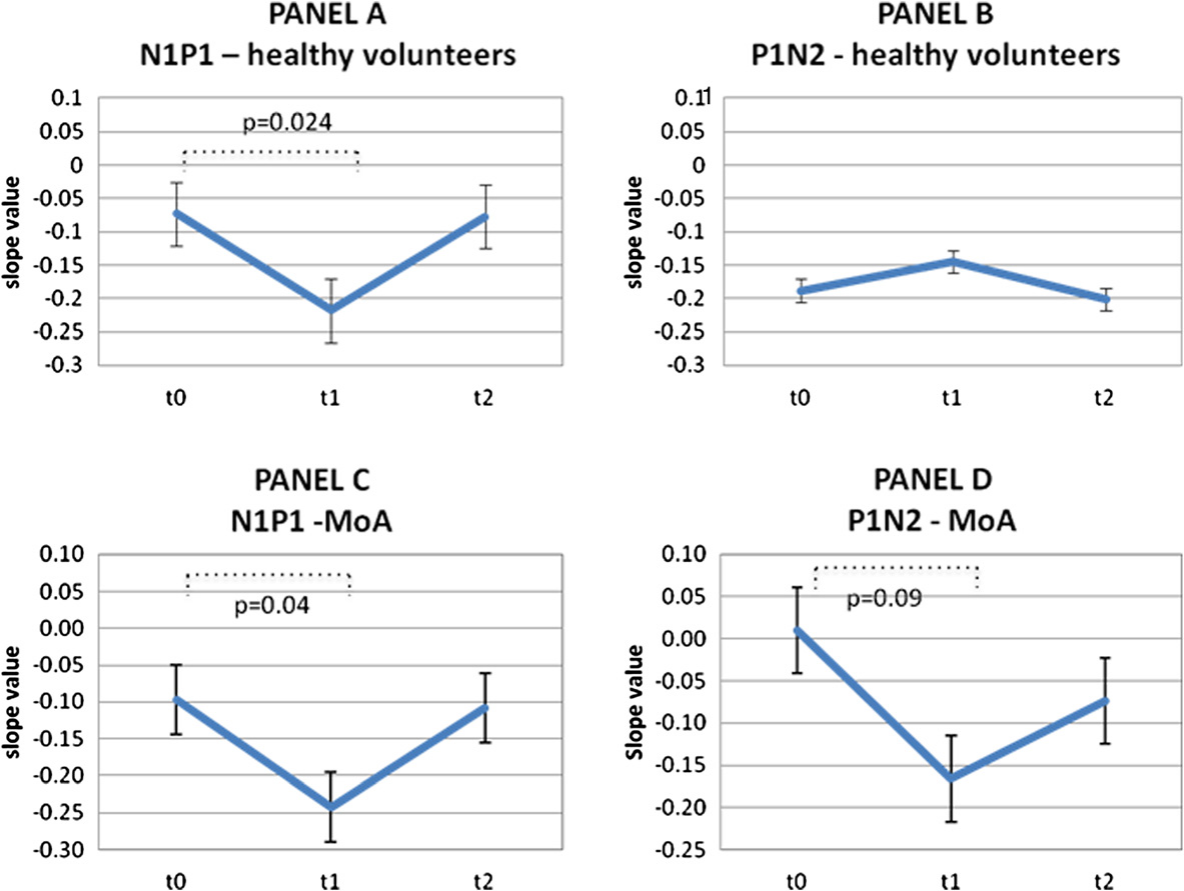
**Time-dependent changes of habituation slope after anodal tDCS.** From the up to the bottom of the table the changes on habituation slopes induced by anodal tDCS on N1P1 and P1N2 in healthy volunteers (HV, Panel **A** and Panel **B**) and episodic migraine patients (MoA, Panel **C** and Panel **D**) at T0, T1 and T2. The habituation value is expressed as the decrement of the response with stimulus repetition so a more negative value of the slope corresponds to a stronger habituation. The value of the slope at T0, T1, T2 was reported at every time point as it is obtained by the interpolation of mean values in all blocks by linear regression equation. The x axis corresponds to the time (T0 = baseline; T1 = immediately after the stimulation; T2 = after 3 hours).

The results of the electrophysiological study are presented in Table 1 and Figure 2.

**Table 1:** This table shows the results of the electrophysiological study: Pattern Reversal-VEP initial amplitudes (N1P1 and P1N2, μV), and habituation slopes in healthy volunteers (HV) and episodic migraineurs (MoA), before, just after and 3 h after anodal tDCS

**Groups and VEP comparison**	**First block amplitude (μV)**			**Habituation slope (over six blocks)**		
	**Before**	**After**	**+ 3h**	**Before**	**After**	**+ 3h**
Healthy volunteers						
(n=11)						
N1P1	6.1±2.0	6.8±2.6	6.3±2.2	-0.07±0.14	-0.21±0.14*	-0.08±0.14
P1N2	6.6±2.1	6.5±2.0	6.0±1.6	-0.18±0.19	-0.14±0.16	-0.12±0.25
Episodic migraineurs						
(n=13)						
N1P1	7.1±2.9	7.3±3.1	7.2±2.7	-0.10±0.11	-0.24±0.18*	-0.11±0.17
P1N2	6.6±2.6	6.4±2.9	6.8±2.5	-0.01±0.21	-0.17±0.24	-0.07±0.21

The * mark corresponds to a significant change (p < 0.05).

In baseline (T0), HV and MoA did not differ in first PR-VEP block amplitude, nor in N1P1 habituation slopes (p > 0.05). However, P1N2 habituation slope was significantly deeper in HV than in MoA (−0.23 in HV vs. -0.05 in MoA; p = 0.04), which mirrors a lack of habituation in MoA compared to HV.

In the HV group, anodal tDCS stimulation had no effect on PR-VEP first block amplitude (N1P1: 6.1 μV ±2.0 at T0 vs. 6.8 μV ±2.6 at T1; p = 0.45; P1N2: 6.6 μV ±2.1 at T0 vs. 6.5 μV ±2.0 at T1; p = 0.49), and did not modify the amplitude of subsequent blocks (Table 1). However, the habituation slope of N1P1 amplitude became more negative after tDCS stimulation, i.e. tDCS was able to strengthen habituation in HV at T1 (p = 0.024, Figure 2 Panel A) but this change in habituation did not persist after 3 hours (T2) where it returned on average to baseline values.

In the MoA group, anodal tDCS did not induce any significant effect on VEP amplitudes as well (Table 1). However, like in HV, N1P1 and P1N2 habituations increased immediately after anodal tDCS (T1), and for N1P1 slope this change was significant ( −0.11 to −0.24 after tDCS, p = 0.04, Figure 2 Panel C), meaning that tDCS was also able to increase the habituation level in MoA. These changes did not last for a long time and returned to baseline at T2 as well.

### Therapeutic study

**Figure 3:**
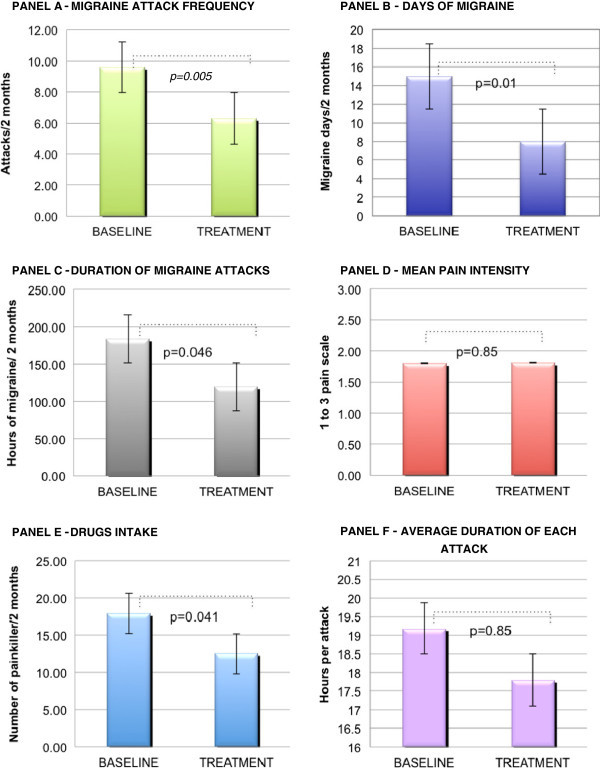
**This figure shows the outcome of the therapeutic pilot trial.** The averages and standard deviations (black lines) of the following clinical parameters are displayed at baseline and for the whole period of tDCS treatment: migraine frequency (Panel **A**), days with migraine (Panel **B**), cumulated duration of all headache attacks (Panel **C**), pain intensity per attack (Panel **D**), acute drug intake (Panel **E**) and duration of each single attack (Panel **F**).

The results of the pilot therapeutic study with anodal tDCS in MoA are presented in Figure 3 and are encouraging.

Hence, during the 8 weeks of anodal tDCS treatment, there was already on average a significant reduction of migraine frequency, which was decreased from 9.6 days in 2 months to 6.3 (34%,p = 0.005), while there was a remarkable reduction in the number of migraine days from 15 to 8 (47%, p = 0.01). The average cumulative attack duration over 2 months decreased from 184 to 119 hours (35%, p = 0.043), and the average acute treatment intake dropped from 18 tablets to 13 in two months (p = 0.041). The duration of each attack slightly decreased as well, but in a non-significant manner (p = 0.70).

We performed a further subanalysis where we only considered the outcome within the last 4 weeks of tDCS, which was compared to the baseline diary of the month preceding tDCS application, on the assumption that the clinical effect would improve with the repetition of tDCS sessions. Migraine frequency reduction was more pronounced during the second month of therapy, with a mean decrease from 5 to 3 attacks (−38%; p = 0.03), the number of migraine days also decreased from 8 to 4.3 (48%, p = 0.002), and noteworthy the average attack duration dropped from 88.5 to 33.2 (60%, p = 0.02). The drug intake tended to decrease from 9 pills/month to 6 pills/month (28%, p = 0.06).

To rule out a pure long-term pharmacological effect of the ongoing preventive therapy we then compared patients with (N = 5) and without (N = 5) migraine preventive treatment. The evolution under tDCS treatment was similar in both groups: patients without drug therapy had a frequency reduction from 9.2 ±1.64 to 5.4 ± 2.19 attacks in two months (p = 0.04), while in patients under preventive therapy the frequency decreased from 10 ± 1.4 to 7.2 ± 1.8 attacks in two months (p = 0.04). No inter-group difference was found (p = 0.45). In addition, when we compared the sustained post-treatment benefit, we found no difference between the 2 groups in terms of attack recurrence: the group without any drug preventive therapy returned to the baseline migraine frequency 4.2 ± 3.8 weeks after the end of tDCS, while the group under prophylaxis returned to baseline migraine frequency after 5.4 ± 3.7 weeks (p = 0.62). Hence, a delayed effect due to the drug preventive treatment seems unlikely.

### Adverse events

No adverse events were reported by patients, neither in the electrophysiological nor in the therapeutical tDCS study, but a light itching sensation that invariantly disappeared in few minutes after the end of stimulation.

## Discussion

As we said before, the lack of cortical habituation to repetitive sensory stimuli is the more reproducible electrophysiological hallmark of the migrainous brain when recordings are made interictally. As far as we know, this is the first study using excitatory tDCS in order to modify habituation, especially to normalize it in migraineurs, and trying afterwards to translate these findings into a new kind of preventive therapy.

### Electrophysiological study

The results of our electrophysiological study are in line with those found previously with rTMS, where an excitatory 10 Hz stimulation was able to increase the initial lower VEP response and restore normal habituation in migraineurs [12]. The latter supported the idea that the habituation deficit could be due to a lower preactivation level of the brain cortex, and suggested that transcutaneous central neurostimulation could have therapeutic potentials in migraine.

We chose to perform anodal, i.e. “excitatory” tDCS along the same line, in order to increase visual cortex preactivation and subsequently correct the lack of habituation in migraineurs. However we did not find any enhancement of the VEP initial amplitude, neither in healthy subjects nor in migraineurs, but surprisingly tDCS increased habituation of the second component of the VEP in both groups. Like in the rTMS [12] the duration of tDCS effect on habituation was brief and VEP recordings performed after 3 hours (T2) demonstrated that habituation slopes had come back to baseline values. The significant increase of habituation in absence of any initial amplitude modification, i.e. any cortical preactivation level enhancement with tDCS, is difficult to explain. It could be attributed to the different mechanisms of action of tDCS and rTMS [23]. Moreover, some authors suggest that the cortical dysexcitability found in migraine could also be related to abnormal inhibitory circuits within the cortex, and that an impaired habituation does not necessary requires a lower preactivation level [35].

The relationship between the electrophysiological abnormalities and the patient clinical state is still obscure and complex; and whether the normalization of electrophysiological responses with neuromodulation could lead to a concomitant significant clinical improvement in migraineurs remains debated. Hence, we had shown a while ago that effective prophylaxis with betablockers was correlated to an average normalization of auditory evoked potentials (AEP), but not effective riboflavine therapy, which did not modify AEP, suggesting 2 distinct mechanisms [36]. In another study we had found similar electrophysiological abnormalities in healthy volunteers with a familial history of migraine, although they did not have any headache themselves at the time of the recordings [37]. A recent publication found that topiramate [38], one of the most effective drugs in migraine prevention, was able to normalize habituation in these patients. At baseline, episodic migraineurs showed a significant lack of habituation, which disappeared after 2 months of treatment with topiramate, and the individual improvement of habituation was positively correlated with the clinical benefit.

This underlined the need for a proof-of-concept clinical trial using a central neuromodulation technique able to normalize habituation, such as anodal tDCS.

### Therapeutic study

The results of our pilot trial with anodal tDCS in only 10 MoA patients are encouraging and most clinical variables already significantly improved within 8 weeks of treatment. Migraine frequency, migraine days, painkillers intake and attack duration decreased, and this improvement was even stronger in the second month of treatment (except for acute medications), which underlines that anodal tDCS preventive therapy sessions should be continued on a regular basis for at least 2 months, like drug prophylaxis or other non-invasive neurostimulation techniques, for example supraorbital nerve stimulation [39]. Migraine days and attack duration exhibited the strongest average improvement with respectively 48% and 60% reduction. However, we are aware that our study has some shortcomings. A placebo effect cannot be ruled out without a randomized controlled trial. Moreover, some patients might have a long-term response to drug prophylaxis, but the comparison between treated and untreated patients could argue against this hypothesis (both responded similarly to tDCS), as well as the attack recurrence observed in most patients after the end of tDCS, within a variable time interval. Finally, the improvement of patients under long-term tDCS therapy contrasts with the results of the electrophysiological study, where one single tDCS session over the visual cortex only induced a very short-term habituation modification (<3 h). However, the repetition of tDCS sessions over 8 weeks could have been responsible for neuroplastic changes and induce sustained modifications within the underlying visual cortex. Unfortunately, we did not record VEPs before and after the 8 weeks of tDCS therapy. These measures could be worthwhile in a next study.

In a pathophysiological point of view, these results emphasize that the lack of habituation is probably playing a key role in the genesis of migraine headache, even if other pathological mechanisms may also be involved.

There are few existing trials on migraine prevention using central non-invasive neurostimulation methods, i.e. rTMS or tDCS, and their stimulation paradigms differed according to the author’s baseline pathophysiological hypotheses. Thus, in order to correct an eventual cortical hyperexcitability, Teepker et al. [30] and Antal et al. [31] applied inhibitory stimulations, respectively 1Hz-rTMS and cathodal tDCS over the vertex and the visual cortex, leading to minor or negative clinical results. This could eventually be due to an incorrect baseline assumption.

Chronic migraine management is often challenging and thus non-invasive neurostimulation could offer a new hope to these patients. The patients included in our clinical study did not fulfill the criteria for chronic migraine, and we stress that excitatory stimulations paradigms could even be counterproductive in these patients. Even if the excitatory 10 Hz-stimulation of the dorsolateral prefrontal cortex (DLPFC), known for its implication in pain control [40], was able to slightly improve chronic migraine patients [29], these results were also uncontrolled and there was a comorbid state of depression which might have been a major confounding factor. Hence, beyond depression, chronic migraine seems to differ from episodic migraine in terms of brain excitability. While habituation deficit is a hallmark of the disease in episodic migraine, in chronic migraine, surprisingly, habituation does not differ from control subjects [19]. Recent works suggest that in chronic migraine, the cerebral cortical excitability increases as the activity of cortical inhibitory interneurons decreases, which finally leads to a normal habituation, at least in visual areas (for details, see [41]). When the same chronic patients are successfully treated and evolve to episodic migraine, the lack of habituation reappears. These data support the idea that chronic migraine could be a “never-ending attack [20,21]. Thus, we believe that chronic migraine should paradoxically be treated using inhibitory stimulations unlike episodic migraine and that excitatory stimulations, like anodal tDCS reported in the present study, could be ineffective or even worsen these patients. More neurostimulation studies are warranted to confirm this assumption.

## Conclusion

This study demonstrates for the first time that a 15-min session of anodal tDCS over the visual cortex is able to transiently increase habituation in healthy volunteers but also in episodic migraineurs. Its mechanism of action does not seem to involve cortical preactivation modifications as the initial amplitude of the visual evoked potentials is not modified.

The same excitatory paradigm applied twice a week during 8 weeks as preventive therapy in 10 episodic migraineurs results in a significant reduction of migraine attack frequency, migraine days, painkiller intake and attack duration. All positive effects seem to improve with time, suggesting that preventive therapy with anodal tDCS should be performed on a regular basis, and could involve additional slow neuromodulating processes.

These encouraging results need to be confirmed in a well-designed randomized controlled trial.

## Competing interests

The authors declare they have no competing interests.

## Authors’ contributions

AV has participated in the study design, has recorded patients, has analyzed the results and has drafted the manuscript. TSD has participated in the recordings of patients and the analysis of the results. SS has participated in the discussion of the results. MA helped AV for the recordings of patients. VDP has participated in the study design and the recordings of patients. AC and VDPi are senior supervisors of AV at the Sapienza University. JS has participated in the design of the study, results discussion and manuscript revision. DM has participated in the design of the study, patients recruitment, results discussion, manuscript drafting and revision. All authors read and approved the final manuscript.
